# Variants in the SMARCA4 gene was associated with coronary heart disease susceptibility in Chinese han population

**DOI:** 10.18632/oncotarget.14387

**Published:** 2016-12-30

**Authors:** Xuan Guo, Xiaohong Wang, Yuan Wang, Chunyan Zhang, Xiaohui Quan, Yan Zhang, Shan Jia, Weidong Ma, Yajie Fan, Congxia Wang

**Affiliations:** ^1^ Department of Cardiovascular Medicine, The Second Affiliated Hospital of Xian Jiaotong University, Xian, Shaanxi 710004, China; ^2^ Department of Cardiovascular Medicine, The First Hospital of Xian, Xian, Shaanxi 710004, China; ^3^ The Department of Orthopedics, Peoples Hospital of Manzhouli City, Manzhouli, Inner Mongolia 021400, China

**Keywords:** SMARCA4, coronary heart disease, gene polymorphisms, case-control study, Pathology Section

## Abstract

**Background:**

Coronary heart disease (CHD) is the leading cause of death worldwide. Many single-nucleotide polymorphisms (SNPs) are found to be related to the risk of CHD in previous studies. This study investigated whether polymorphism of *SMARCA4* gene is associated with CHD.

**Materials and methods:**

Genotypes at five CHD-relevant SNPs were determined in 456 cases of incident CHD and 685 unaffected controls in Chinese Han population using ?^2^ test, genetic model analysis and haplotype analysis. We also analysis the differences in continuous variables among the subjects with three genotypes of related genes were assessed using the ANOVA.

**Results:**

We identified two susceptibility SNPs in the *SMARCA4* gene that were potentially associated with a decreased risk of CHD. We identified rs11879293 (OR, 0.74; 95% CI, 0.59-0.96; *P* = 0.012) and rs12232780 (OR, 0.70; 95% CI, 0.54-0.90; *P* = 0.005) were associated with a decreased risk of CHD risk under the log-additive model adjusted by gender and age. Meanwhile, we also found that significant differences in glucose concentrations with rs11879293 and rs1122608 different genotype. Serum LDL-C and HDL-C were seen among the 3 genotypes of rs12232780 exist differences.

**Conclusion:**

This study provides an evidence for polymorphism of *SMARCA4* gene associated with CHD development in Chinese Han population.

## INTRODUCTION

Coronary heart disease (CHD) is a leading cause of death worldwide. In particular, CHD results in 700 000 deaths in China and 502 000 deaths in the United States annually [1, 2]. CHD is a common complex disease that both genetic factors and lifestyle contribute to the demonstration of the disorder. A significant proportion of CHD is explained by risk scores based on age, gender and modifiable risk factors such as blood lipid profile [3]. Meanwhile, CHD is considered to be approximately 40-50% heritable and family history is a predictive factor of CHD [4]. Apart from monogenic disease, genetic information is not resulted in appreciable improvements in prediction over non-genetic risk factors. Pharmacologic preventive therapies are aimed at those at high risk > 20% 10-year risk of CHD. Using risk scores, 15-20% of myocardial infarction (MI) patients would be considered from a substantial population attributable fraction of CHD [5]. According to previous reports, genome-wide association studies (GWAS) have successfully identified a lot of genetic mutations and chromosomal loci that are associated with CHD [6–9].

The *SMARCA4* gene encodes an ATP-dependent helicase BRG1 and it belongs to SWI/SNF (switching defective/sucrose nonfermenting) complex [10]. According to previous researches, *SMARCA4* is one of the most broadly mutated subunits, developing an understanding of the mechanisms by which mutation of *SMARCA4* drives cancer and of the vulnerabilities created carries major disease relevance [11]. Fujimaki et al. [12] performed a case-control study to reveal the relation between *SMARCA4* genetic polymorphisms and coronary artery disease (CAD) in a Japanese population. Jamaldini et al. [13] found intron 30 of *SMARCA4* gene include rs1122608 was associated with CAD in Iranian population. Previous studies also identified rs11879293, rs12232780, rs2072382 and rs1529729 variants effected on lipid parameters and dyslipidemia related disease [12, 14, 15]. However, the role of *SMARCA4* gene mutation in the development of CHD remains poorly understood in Chinese Han population. The primary aim of this analysis was to determine whether exist the relationship between polymorphisms of *SMARCA4* gene and CHD in the Chinese Han population.

**Table 1:** Basic characteristics of this study population

Parameters	Cases	Controls	*p*
No.	456	685	0.010
Males	291(63.8%)	385(56.2%)	
Females	165(36.2%)	300(43.8%)	
Mean age	61.17±11.86	48.59±9.56	*p* <0.001
ALT (U/L)	31.15±2.11		
AST (U/L)	36.41±2.93		
GGT (U/L)	44.65±3.77		
TP(g/L)	66.39±0.3		
GLU (mmol/L)	6.34±0.11		
TG (mmol/L)	1.8±0.07		
TC (mmol/L)	4.09±0.06		
HDL-C (mmol/L)	1.13±0.01		
LDL-C (mmol/L)	1.92±0.04		
APOA1 (g/L)	1.26±0.01		
APOB (g/L)	1±0.02		
Lp(a) (mg/L)	239.2±12.14		
PLT (109/L)	169.42±3.56		
PCT (%)	1.14±0.16		
MPV (fl)	13.08±0.35		
PDW (%)	14.23±0.15		

ALT: alanine aminotransferase, AST: aspartate aminotransferase, GGT: gamma-glutamyl transpeptidase, TP: total protein, GLU: glucose, TG: triglyceride, TC: total cholesterol, HDL: high-density lipoprotein, LDL: low-density lipoprotein, apoA: apolipoprotein A, apoB: apolipoprotein B, LP(a): lipoprotein, PLT: platelet, PCT: plateletcrit, MPV: Mean Platelet Volume PDW: platelet distribution width. *p* < 0.05, statistical significance

## RESULTS

### Base information

We included a total of 456 CHD patient cohorts consisted of 291 males and165 females with a mean age of 61.17 ± 11.86 years at the time of diagnosis. The 685 healthy controls included 385 males and 300 females with a mean age of 48.59 ± 9.56 years at the time of study enrollment. We found no differences between gender or age distribution between the case and control groups. The characteristics of these patient cohorts are summarized in Table 1. Five SNPs in the *SMARCA4* gene were analyzed in this study. Chromosomal, position, band, HWE *p* value, alleles A/B, MAF control and MAF case for all the SNPs are shown in Table 2. The rs2072382 were cut off at 5% HWE *p* level.

**Table 2:** Basic SNPs in *SMARCA4* gene summary of all study participants

SNP ID	Alleles A/B	Chromosome	Position	Band	HWEs *p* value	MAF control	MAF case	OR(95%CI)	*p* value
rs11879293	A/G	19	11072610	19p13.2	0.5577	0.266	0.240	0.87(0.72-1.06)	0.17
rs12232780	A/G	19	11132080	19p13.2	0.2722	0.224	0.194	0.83(0.68-1.03)	0.09
rs2072382	T/C	19	11152650	19p13.2	0.0100*	0.279	0.326	1.25(1.04-1.50)	0.02
rs1529729	C/T	19	11163562	19p13.2	0.1273	0.228	0.220	0.96(0.78-1.17)	0.69
rs1122608	T/G	19	11163601	19p13.2	0.256	0.093	0.084	0.90(0.67-1.21)	0.50

A/B stands for minor/major alleles on the control sample frequencies.

SNPs are excluded at 5% HWE *P* level

*p* < 0.05 indicates statistical significance

*p* < 0.01 indicates statistical significance for multiple comparison

### SMARCA4 gene and CHD

Using logistic test, four models (recessive, dominant, codominant and log-additive) were applied for analyzing the association of SNPs and CHD risk by listed in Table 3. We identified rs11879293 was associated with a decreased risk of CHD risk under the log-additive model (OR, 0.74; 95% CI, 0.59-0.96; *P* = 0.012) adjusted by gender and age. We also identified rs12232780 was associated with a decreased risk of CHD risk under the log-additive model (OR, 0.70; 95% CI, 0.54-0.90; *P* = 0.005) adjusted by gender and age. Used multiple comparison, eight tests were performed, a significance level was *P* < 0.0063(0.05/8) based on Bonferroni correction, we found that rs12232780 in *SMARCA4* gene influence the risk of Coronary heart disease.

**Table 3:** Association of SNPs with coronary heart disease risk based on logistical tests

SNP	Model	Genotype	control	case	crude analysis		adjusted analysis	
					OR (95% CI)	*P*-value	OR (95% CI)	*P*-value
rs11879293	Codominant	G/G	365 (53.4%)	262 (57.5%)	1	0.380	1	0.041
		G/A	273 (40%)	169 (37.1%)	0.86 (0.67-1.11)		0.73 (0.54-0.99)	
		A/A	45 (6.6%)	25 (5.5%)	0.77 (0.46-1.29)		0.57 (0.31-1.04)	
	Dominant	G/G	365 (53.4%)	262 (57.5%)	1	0.180	1	0.016
		G/A-A/A	318 (46.6%)	194 (42.5%)	0.85 (0.67-1.08)		0.71 (0.53-0.94)	
	Recessive	G/G-G/A	638 (93.4%)	431 (94.5%)	1	0.440	1	0.140
		A/A	45 (6.6%)	25 (5.5%)	0.82 (0.50-1.36)		0.65 (0.36-1.17)	
	Log-additive	---	---	---	0.87 (0.72-1.06)	0.160	0.74 (0.59-0.94)	0.012
rs12232780	Codominant	G/G	406 (59.4%)	292 (64%)	1	0.200	1	0.019
		G/A	248 (36.3%)	151 (33.1%)	0.85 (0.66-1.09)		0.72 (0.53-0.97)	
		A/A	29 (4.2%)	13 (2.8%)	0.62 (0.32-1.22)		0.43 (0.19-0.98)	
	Dominant	G/G	406 (59.4%)	292 (64%)	1	0.120	1	0.011
		G/A-A/A	277 (40.6%)	164 (36%)	0.82 (0.64-1.05)		0.69 (0.51-0.92)	
	Recessive	G/G-G/A	654 (95.8%)	443 (97.2%)	1	0.210	1	0.072
		A/A	29 (4.2%)	13 (2.8%)	0.66 (0.34-1.29)		0.49 (0.22-1.09)	
	Log-additive	---	---	---	0.83 (0.67-1.02)	0.078	0.70 (0.54-0.90)	0.005

*p* < 0.05 indicates statistical significance

*p* < 0.0063 indicates statistical significance for multiple comparison

### SMARCA4 gene and clinical information

The basic characteristics of the study subjects stratified by different genotype are shown in Table 4. We also found significant differences in glucose concentrations with *SMARCA4* rs11879293 and *SMARCA4* rs1122608 different genotype. For rs11879293, the mean (± Std) serum glucose concentration was 6.18 ± 2.21mmol/L for the GG genotype (lowest), 6.36 ± 2.17mmol/L for the GA genotype, 7.79 ± 4.82 mmol/L for the AA genotype (highest) (*p* = 0.006). For rs1122608, the mean (± Std) serum glucose concentration of GG, GT and TT is 6.20 ± 2.13 mmol/L, 7.08 ± 3.60mmol/L, and 6.65 ± 1.45mmol/L, respectively. Subjects carrying the T allele of *SMARCA4* rs1122608 had higher glucose concentration (*p* = 0.019)

**Table 4:** Clinical and biochemical characteristics of participants, stratified by SMARCA4 gene polymorphism status

SNP	Genotype			*p*
rs11879293	GG	GA	AA	
GLU	6.18±2.21	6.36±2017	7.79±4.82	0.006
TC	3.99±1.04	4.29±1.30	3.96±1.28	0.033
LDL-C	1.80±0.65	2.12±1.07	1.84±0.69	0.001
rs12232780	GG	GA	AA	
HDL-C	1.13±0.25	1.14±0.26	0.95±0.14	0.047
LDL-C	1.84±0.69	1.84±0.69	1.67±0.58	0.012
rs1529729	TT	TC	CC	
PDW	14.38±3.03	13.85±2.71	15.63±3.08	0.043
rs1122608	GG	GT	TT	
GLU	6.20±2.131	7.08±3.60	6.65±1.45	0.019

*p* < 0.05 indicates statistical significance

*p* < 0.0071 indicates statistical significance for multiple comparison

Significant differences in serum LDL-C and HDL-C were seen among the 3 genotypes of rs12232780. The mean serum LDL-C was 1.84±0.69 mmol/L in the GG group, 1.67 ± 0.58 mmol/L in the AA group (*p* = 0.012). Subjects carrying the G allele of 12232780 had higher serum HDL concentrations (*p* = 0.047): 1.13±0.25 mmol/L for the GG genotype, 1.14±0.26 mmol/L for the GA genotype, 0.95±0.14 mmol/L for the AA genotype.

We also found that PDW exist differences among the three genotypes of rs1529729 (*p* = 0.043): 14.38±3.03 for the TT genotype, 13.85±2.71 for the TC genotype, 15.63±3.08 for the CC genotype.

Used multiple comparison, seven tests were performed, a significance level was *P* < 0.0071(0.05/7) based on Bonferroni correction, significant differences in serum LDL-C and glucose concentration were seen among the three genotype of rs11879293 in *SMARCA4* gene.

## DISCUSSION

In a case-control study of incident CHD within the population based Chinese Han cohort, we evaluated the associations of the disease with four SNPs documented as associated with CHD. We identified that rs11879293 and rs12232780 in the *SMARCA4* gene was associated with a decreased risk of CHD. Meanwhile, we also found that significant differences in glucose concentrations with rs11879293 and rs1122608 different genotype. Serum LDL-C and HDL-C were seen among the 3 genotypes of rs12232780 exist differences.

In our research, we found that *SMARCA4* rs11879293 and rs12232780 seemed to have strong protective effects on the CAD. At present, there were no relevant reports on the rs11879293 and rs12232780 and coronary heart disease. *SMARCA4* is located in chromosomal region of 19p13.2, and its protein is the important catalytic component of the SWI/SNF complexes. It is composed of multiple domains, a conserved C-terminal bromodomain, the less characterized N-terminal region which have crucial effect on DNA binding, recruitment of SWI/SNF and the recognition of modified histone proteins [16]. *SMRACA4* is located closely to the low-density lipoprotein receptor gene and disrupting chromatin structure regulates the transcription of various genes using the chemical energy of adenosine triphosphate hydrolysis [17]. Besides, we rarely know that the functional meaning of this marker of the 19p13 locus, but being associated with LDL-cholesterol levels, pathogenic SNP within the adjacent *LDLR* could be in linkage disequilibrium with the 19p13 locus [18].

Pan H. et al. [19] found that rs11879293 associated with the risk of hepatocellular carcinoma is more pronounced in males, younger individuals ( < 60 y), and nondrinkers. However, we did not found rs11879293 associated with CHD by previous reports. Amankwah et al. [20] provide suggestive evidence that rs12232780 in *SMARCA4* variant in the chromatin remodeling machinery may be associated with an increased risk for oligodendroglioma. We all know that coronary heart disease risk factor consists of LDL-C elevated and HDL-C decreased. We also found that LDL-C exist difference among the 3 genotypes in rs11879293 and rs12232780. The level of LDL-C in mutation genotype was lower than the wild type, and therefore, we hypothesized that these rs11879293 and rs12232780 might affect the occurrence of coronary heart disease by affecting the level of LDL-C.

In 2009, Knouff et al. [21] discovered that rs1122608 was associated with myocardial infarction. This site is a high risk factor of myocardial infarction in the Russian population, but the correlation with myocardial infarction in Han population is not obvious [22]. This loci rs1122608 was associated with LDL-C and coronary heart disease in Caucasian population [18]. In the research literature in Iran also suggested that rs1122608 (GT or TT) associated with the severity of coronary heart disease [13]. While, in our research, no significant result exist between rs1122608 polymorphism and Coronary heart disease. In future studies, we will consider that the *SMARCA4* gene may function differently in varying disease mechanisms.

There were several limitations to our study. First, all the samples were from the Chinese Han population living in Xi’an city or its surrounding area and from the same hospital. There were a substantial number of confounding factors that may have caused type I errors (false-positive results) in our association study. Second, our samples included 456 cases and 685 healthy controls and we performed a power analysis that showed that the power of four SNPs was < 0.75. Our samples included 456 cases and 685 healthy controls and we performed a power analysis that showed that the power of four SNPs was < 0.75. It is generally believed that the statistical validity of a study should be more than 75%. If the sample size is too small will reduce the degree of confidence, that is, the effectiveness of the reduction, if not up to the standard results are not credible. To obtain a statistical power of 80%, 3657 subjects with CHD and 5485 controls are required for rs11879293, and 2402 subjects with CHD and 3604 controls for rs12232780. At present, so many samples (3657 subjects with CHD and 5485 controls) are difficult to collect, and it is not possible to collect so many samples in the short term. In our research, it exist an issue of the small study population, and the low statistical power. However, we will continue to collect coronary heart disease and healthy population, increase the statistical power, and continue to study the effect of SMARCA4 gene on coronary heart disease

BMI is an important cardiovascular disease risk factor, and raised blood pressure, cholesterol, and glucose partly mediate its effects. Beyond that, smoking, hypertension, diabetes mellitus, dyslipidemia, chronic kidney disease, and obesity are also risk factors for coronary heart disease. So, these characteristics are important for case-control studies. But in our study, we mainly research the association between gene polymorphism and coronary heart disease, and analysis the influence of gene polymorphism on biochemical index. In the process of collecting samples, participants did not provide us with the information (e.g. smoking, hypertension, diabetes mellitus, dyslipidemia, chronic kidney disease, and obesity). But in the future, we will continue to collect samples, perfect the clinical information, comprehensive study of the impact of polymorphism on coronary heart disease.

In conclusion, our comprehensive analysis of SNPs in the *SMARCA4* gene suggests that *SMARCA4* genotypes and haplotypes are associated with CHD risk in Chinese Han population. In the future, we will continue to collect samples, perfect the clinical information, comprehensive study of the impact of polymorphism on coronary heart disease.

## MATERIALS AND METHODS

### Ethics statement

The protocol in this study conformed to the principles of the Declaration of Helsinki and was ratified by the Ethical Committee of the Second Affiliated Hospital of Xi’an Jiaotong University Health Science Center, China. Signed informed consent was obtained from each participant.

### Study participants

In our study population, all analyses were restricted to Chinese Han population. We recruited a total of recently diagnosed confirmed 456 CHD patients from September 2012 to November 2014 into an ongoing molecular epidemiological study at the Second Affiliated Hospital of Xi’an Jiaotong University Health Science Center in Xi’an city, China. There were no age, sex, or disease stage restrictions for case recruitment. All patients were recently diagnosed confirmed to have CHD. Data collection for identification and validation of coronary events at baseline and throughout follow-up included the World Health Organization (WHO) chest pain questionnaire, Electrocardiogram(ECG)(coded using Minnesota Classification Code), self-reported doctor diagnosis of disease, record linkage to hospital discharge data and death certificates, and scrutiny of general practitioner records [23]. Questionnaire data were collected on history of diabetes blood pressure medication use, daily smoking, history of MI at baseline and family history of CHD [24]. In the current analyses, the classification of CHD was based on validated events and comprised of fatal or non-fatal myocardial infarction (MI), coronary artery bypass surgery, angioplasty, angina and/or unspecified ischaemic heart disease. We also collected the clinical data of CHD patient, including PLT, PCT, MPV, PDW, and Serum concentrations of ALT, AST, GGT, TP, GLU, TG, TC, HDL-C, LDL-C, APOA, APOB, Lp(a).

We also selected 685 healthy unrelated individuals between November 2012 and August 2014 from the medical examination center, the Second Affiliated Hospital of Xi’an Jiaotong University Health Science Center, who were recruited for scientific research into human complex diseases such as lung, stomach and liver chronic diseases. All the participants were restricted to Chinese Han who lived in Xi’an city and its surrounding areas.

### SNP identification

We genotyped five single nucleotide polymorphisms (SNPs) with minor allele frequency (MAF) > 5% in *SMARCA4* genes in the HapMap Asian population [12]. 5 ml venous blood was collected in a tube containing EDTA, and genomic DNA was extracted fromleukocytes using the GoldMag nanoparticles method (GoldMag Ltd. Xi′an, China) according to the manufacturer's instructions. DNA concentration was measured by spectrometry (DU530 UV/VIS spectrophotometer; Beckman Instruments, Fullerton, CA, USA). We designed the Multiplexed SNP Mass EXTEND assay using Sequenom MassARRAY Assay Design version 4.0 software [25].

### Statistical analysis

All continuous data are presented as means ± standard deviations (SDs). Student t test were used to compare the distribution of continuous variables. Differences in continuous variables among the subjects with three genotypes of related genes were assessed using the ANOVA. Statistical analyses were performed using SPSS 19.0 statistical package (SPSS, Chicago, IL). The genotype frequencies of each SNP in the control subjects were checked using the Hardy-Weinberg equilibrium (HWE). Power analysis was carried out using the online calculator at http://sampsize.sourceforge.net/iface/s3.html. Data analysis was performed using SPSS version 16.0 statistical package (SPSS, Chicago, IL, USA) and Microsoft Excel. The significance of the difference of alleles and genotype frequencies between the groups was tested using the chi-square method [26]. *P* < 0.05 was considered to represent statistical significance. Differences in the distribution were analyzed using logistic regression. Odds ratios (ORs) and 95% confidence intervals (CIs) were tested using unconditional logistic regression analysis with adjustment for age and gender [27]. We also performed Bonferroni correction to assess the effect of genetic polymorphisms on coronary heart disease. Finally, we used the SHEsis software platform (www.nhgg.org/analysis/) for analyses of LD, haplotype construction, and genetic association at polymorphism loci [28].
